# Effect of Different Rates of Nitrogen Fertilization on Crop Yield, Soil Properties and Leaf Physiological Attributes in Banana Under Subtropical Regions of China

**DOI:** 10.3389/fpls.2020.613760

**Published:** 2020-12-21

**Authors:** Jianbo Sun, Wenbin Li, Chunqiang Li, Wenjun Chang, Shiqing Zhang, Yanbo Zeng, Changying Zeng, Ming Peng

**Affiliations:** ^1^Key Laboratory of Biology and Genetic Resources of Tropical Crops, Ministry of Agriculture, Institute of Tropical Bioscience and Biotechnology, Chinese Academy of Tropical Agricultural Sciences, Haikou, China; ^2^College of Tropical Crops, Hainan University, Haikou, China

**Keywords:** nitrogen application rate, soil properties, leaf physiology, crop yield, factor analysis, path analysis

## Abstract

Excessive nitrogen (N) application is widespread in Southern China. The effects of N fertilization on soil properties and crop physiology are poorly understood in tropical red loam soil. We conducted a field experiment to evaluate the effect of nitrogen fertilization rates on physiological attributes (chlorophyll, plant metabolic enzymes, soluble matters) on banana leaves, soil properties (soil enzymes, soil organic matter (SOM), soil available nutrients) as well as banana crop yield in a subtropical region of southern China. The N rates tested were 0 (N_0_), 145 (N_145_), 248 (N_248_), 352 (N_352_), 414 (N_FT_), and 455 (N_455_) g N per plant. The correlations among soil factors, leaf physiological factors and crop yield were evaluated. The results indiated that the high rates of N fertilization (N_FT_ and N_455_) significantly decreased soil available potassium (K) content, available phosphorus (P) content, glutamine synthetase (GS) activity, and soluble protein and sugar contents compared with lower N rates. The N_352_ treatment had the highest crop yields compared with higher N rates treatments, followed by the N_455_ treatment. However, there were no significant differences in crop yields among N fertilization treatments. Factor analysis showed that the N_352_ treatment had the highest integrated score for soil and leaf physiological factors among all treatments. Moreover, the N_352_ treatment was the most effective in improving carbon and nitrogen metabolism in banana. Crop yield was significantly and positively linearly correlated with the integrated score (*r* = 0.823, *p* < 0.05). Path analysis revealed that invertase, SOM and sucrose synthase (SS) had a strong positive effect on banana yield. Canonical correspondence analysis (CCA) suggested that available K, invertase, acid phosphatase and available P were the most important factors impacting leaf physiological attributes. Cluster analysis demonstrated distinct differences in N application treatment related to variations in soil and leaf factors. This study suggested that excessive N fertilization had a negative effect on soil fertility, crop physiology and yield. The lower N rates were more effective in improving crop yield than higher rates of N fertilization. The N rate of 352 g N per plant (N_352_) was recommended to reduce excess N input while maintaining the higher yield for local farmers’ banana planting.

## Highlights

- Reduced N application rate has been proved to be beneficial for both soil and leaf physiological factors.
- The N_352_ treatment had the highest integrated score and yield across all treatments.
- Soil organic matter and metabolic enzymes including invertase and sucrose synthase were the dominant factors affecting yield.
- Acid phosphatase and available potassium were the prominent indicators affecting the leaf physiological attributes.

## Introduction

Nitrogen is an essential mineral nutrient for crop growth and yield (Xu et al., 2012). During the past three decades, N application rates have increased rapidly and excessive quantities of N fertilizers have been used to enhance crop yields. However, Excessive N application could lead to soil acidification as well as worsen the soil environment thus, ultimately has a negative impact on crop growth and yield (Guo et al., 2010; Schroder et al., 2011). Previous studies indicated that reducing N application rates to a reasonable level in maize and wheat planting caused no loss of yield and even small increases (Zhang et al., 2015). Zhao et al. (2014) found that the application of lower N rates sustained high yields compared with higher N rates. Yield reductions in crops with high N fertilization are primarily caused by physiological disorders associated with excessive uptake of N and soil degradation (Qiao et al., 2012). Although, optimum N rates are affected by many factors, studies have shown that a moderate reduction in N inputs does not lead to a decrease in crop yield (Luo Z. et al., 2018) but, conversely, improved N use efficiency (Zhang et al., 2015a).

Excessive N fertilization has caused low N use efficiencies and serious environmental problems (Cui et al., 2016; Zhu et al., 2016). Therefore, rational N fertilization strategies must be considered for achieving high crop production and sustainable agroecosystem.

Nitrogen fertilization can significantly affect soil properties. Soil factors are closely associated with soil nutrient cycling and plant nutrient uptake, and therefore affect productivity. Urease, phosphatase and invertase play key roles in soil N, P, and C cycles (Zhao et al., 2009). These enzyme activities are directly involved in various biochemical reactions in the soil. Soil available nutrients can be directly absorbed by crops and contribute to soil fertility. Soil organic matter (SOM) is responsible for some important soil processes such as soil respiration, soil aggregate stability and water holding capacity (Herencia et al., 2011). These soil properties are considered as important factors determining soil quality (Gong et al., 2015).

Nitrogen fertilization can also affect plant physiological characteristics such as chlorophyll concentration, plant metabolic enzyme activities and soluble proteins, and ultimately, crop production was affected. Leaf chlorophyll concentration is an important photosynthetic capacity attributes for monitoring the N status of plants. Nitrate reductase (NR) and glutamine synthetase (GS) are key enzymes in nitrogen assimilation (Kichey et al., 2006). Sucrose synthase (SS) and sucrose phosphate synthase (SPS) are essential components involved in sucrose to starch conversion (Yang et al., 2004), and their activity can be linked to sink strength and crop yield subjected to N fertilization.

Understanding the effects of N fertilization on soil and crop factors can help with developing strategies to explore appropriate N application rates, and therefore improve crop yields and environmental sustainability.

Red soils are distributed in tropical and subtropical regions of China and are considered an important arable land resource. Banana is one of the most important food crops in tropical and subtropical regions. To meet the needs of increasing crop production and intensive agriculture, excessive N application is still widespread in these regions (Yang et al., 2015; Hu et al., 2016). These N management practices have led to nitrate leaching (Zhou et al., 2016) and negative impacts on soil fertility (Guo et al., 2010; Zhu et al., 2018). As a result, crop growth and production are limited.

The response of soil properties and crop physiological attributes to N fertilization depends on soil type, climate conditions and other factors (Lupwayi et al., 2012; Giacometti et al., 2013). Therefore, optimal N fertilization strategies must be based on specific site and conditions. Various studies have focused on the single effects of N fertilization on soil properties or crops physiology. However, little is known about the correlations among soil properties, banana physiological factors and crop yield under different N application rates, especially in a tropical red loam soil. Effective N management strategies should ensure high crop yield and environmental sustainability. In our previous studies, the P and K application rate for banana production were optimized. To determine an optimal regional N application rate, we assessed the effects of soil properties, leaf physiology and crop yield in banana under different N application rates based on optimal P and K application rate.

Factor analysis is regarded as a typical multivariate statistical method for grouping complex inter-correlating variables into a few interpretable independent factors, and revealing major and distint regularities among the original variables. This is done by extracting latent factors based on weight that better explain the underlying processes responsible for variations (Cesar et al., 2014; Sun et al., 2018). Path analysis is an extension of multiple regression and has been used to quantify the relative contribution of the direct and indirect effects of predictor variables on the dependent variables in many ecosystems (Sackett et al., 2013). A path coefficient represents the magnitude of the direct influences of the independent variable on dependent variable (Barberán et al., 2014). In this study, we carried out path analysis to assess the relative importance of soil and leaf factors on banana yield.

The objectives of this study were (1) to determine the effects of N application rate on soil properties and leaf physicochemical characters; (2) to identify the main soil and leaf physicochemical factors impacting banana yields; (3) to explore the important soil properties impacting leaf physiology; and (4) to estimate the optimal N application rate by examining crop yields and the integrated score for soil and leaf physiological factors.

## Materials and Methods

### Field Site and Experimental Design

The field experiment was performed at the experimental station of the Chinese Academy of Tropical Agricultural Sciences, Haikou (19°51′N, 110°5′E), China. The region is characterized by a typically subtropical monsoon climate with an annual mean temperature of 24°C. The annual average precipitation is 1632 mm, and the average evaporation is 1734 mm. The average monthly minimum and maximum temperature is 15.4 and 33.1°C, respectively. In the study area, the vegetation is dominated by banana, mango and pineapple. The soils are classified as Udic Ferralsol based on the World Reference Base for Soil Resources (FAO, 2015). The dominant parent material of soil is granite. The main soil type is montane lateritic soil. The experimental soil had sandy loam texture. The initial soil (0–20 cm) properties were as follows: SOM content of 1.84 %, soil pH of 5.77, soil available N, P and K of 30.33, 25.55, and 26.42 mg kg^–1^, respectively.

Prior to the start of the experiment, the plots were idle lands without any crops on land. Field experiments were conducted from October 2016 to August 2018 successive cropping seasons, and experimental data were collected in last cropping season. The plantlets of banana with 8 leaves were transplanted into the field in October. A randomized complete block design was established for the variable factor of N rate.

The chemical fertilization rate used by local farmers was: N, 414 g; P_2_O_5_, 150 g; K_2_O, 720 g per plant and was named N_FT_ in this study. Six different rates of N fertilization treatments were 0 (N_0_), 145 (N_145_), 248 (N_248_), 352 (N_352_), 455 (N_455_) g N per plant and N_FT_. In addition, a no fertilization treatment was designed as control (T_CK_). Each treatment was replicated three times. The size of each plot was 90 m^2^ (3 m × 30 m) with a plant population density of 15 plants per plot.

Each plot was fertilized with 10 kg common sheep manure per plant as basal fertilizer. The organic fertilizer contained 74% organic matter, 3% total amino acids and small molecular peptides, 1.2% N, 1.1% P_2_O_5_, and 0.7% K_2_O. The organic fertilizer was spread evenly on the soil surface and immediately incorporated into the plowed soil. Chemical N, P and K fertilizers were applied as urea, calcium superphosphate and potassium sulfate, respectively. All N treatments except N_FT_ were fertilized with 0.185 kg calcium superphosphate and 1.2 kg potassium sulfate per plant. As for N_FT_, the rates of calcium superphosphate and potassium sulfate were 0.25 and 1.33 kg, respectively. NPK application was split 3:4.5:2.5 for the seedling, flowering and budding stage. Chemical N, P, and K fertilizers were incorporated and applied with an independent drip fertigation system. Field practices such as field tillage and irrigation were managed in accordance with local agronomic practices, and weeds were controlled manually. During the growing season, no pesticides were applied.

### Soil Sampling and Analysis

Soil samples were collected from the plow layer (0–20 cm) at the budding stage in early July 2018. Fresh samples were taken from 10 cores within each plot and were mixed thoroughly as one composite sample for later analysis. Soil samples were sieved through a 2-mm mesh and separated into two parts. One part was stored at 4°C for determining soil enzyme activity. The other part was air dried for soil chemical analysis.

Soil urease activity was determined as described by Crowther and Large (1956). A 5 g fresh soil sample was incubated at 37°C for 24 h with 10 ml of 10% (w/v) urea solution and 20 ml of citrate buffer (pH 6.7). The reaction mixture was then diluted with 38°C water to 50 ml and filtered. A 1.0 ml of filtrate was diluted to 10 ml and then treated with 4 ml sodium hydroxybenzene solution and 3 ml of sodium hypochlorite solution for 20 min at room temperature. The released ammonium was quantified in an ultraviolet spectrometer subsystem at 578 nm. Urease activity was expressed as mg NH_4_-N⋅g^–1^ soil⋅24 h^–1^. Invertase activity was determined using the 3,5-dinitrosalicylic acids (DNS) colorimetric method (Wang et al., 2009). A sample of 5 g soil was incubated at 37°C for 24 h with 15 ml of 8% sucrose and 5 ml of phosphate buffer (pH 5.5). A 1.0 ml of filtrate was treated with 3 ml of DNS, and was placed into a boiling water bath for 5 min. The reacted solution was measured at 508 nm and invertase activity was expressed as mg glucose⋅g^–1^ soil⋅24 h^–1^. Acid phosphatase activity was determined using the disodium phenyl phosphate colorimetric method (Tabatabai and Bremner, 1969). A sample of 1 g soil was incubated with 4 ml of 0.2 M modified universal buffer (pH 6.5) and 1 ml of 25 mM p-nitrophenyl phosphate (PNP). After incubation at 37°C for 1 h, the enzyme reaction was stopped by adding 1 ml of 0.5 M CaCl_2_ and 4 ml of 0.5 M NaOH. The suspensions were centrifuged and filtered. Absorbance was measured spectrophotometrically at 400 nm. Enzyme activity was expressed as mg PNP⋅g^–1^ soil⋅24 h^–1^. Soil available N content was determined using the NaOH-hydrolyzing, NH_3_-diffusing and H_3_BO_3_-absorption method described by Subbiah and Asija (1956). Soil available P was extracted with 0.5 M NaHCO_3_ and quantified by molybdenum blue colorimetry (Olsen et al., 1954). Soil available K was extracted with 1.0 M NH_4_OAc and was measured by flame absorption spectrometry (Richards, 1993). Soil SOM content was determined by the potassium dichromate oxidation method (Nelson and Sommers, 1982).

### Leaf Sampling and Analysis

Leaf samples were collected simultaneously with soil samples. The upper fully expanded functional leaf on the main stem was chosen and the middle-upper part was sampled. These samples were placed immediately in liquid nitrogen containers for further analysis. Total of six plants were measured in each plot and the mean values were calculated.

Nitrate reductase activity was assayed as described by Vijayalakshmi et al. (2015). Briefly, a 0.5 g of leaf tissue was added to 2.5 ml potassium phosphate buffer containing 5 mmol cysteine hydrochloride, 1 mmol EDTA and 1 mmol DTT. The 0.3 ml of supernatant was incubated with 0.1 mol potassium phosphate buffer, 0.1 mol potassium nitrate and NADH solution. The reaction was stopped by the addition of zinc acetate. Nitrite was measured at 540 nm by adding sulphanilamide and N1-naphtylethylene-diamine-dihydrochloride. NR activity was expressed as μmol NO_2_^–^⋅g^–1^⋅h^–1^ FW (fresh weight). For GS assay, a 0.5 g leaf tissue was extracted with 3 ml of Tris-buffer containing 0.765 g of Tris–HCl, 0.11 g of cysteine hydrochloride, 0.246 g of MgSO_4_⋅7H_2_O and 0.02 g of EDTA. The enzyme extract was incubated with Tris–HCl buffer, ATP, sodium glutamate, MgSO_4_, L-cysteine, and hydroxylamine. After incubation, 10% FeCl_3_, 20% TCA and 50% HCl were added to the mixture. Glutamyl hydroxamate was measured at an absorbance of 540 nm. GS activity was expressed as μmol γ-glutamyl hydroxamate⋅g^–1^⋅h^–1^ FW (Vijayalakshmi et al., 2015). SPS and SS activities were determined according to the method as described by Hu et al. (2015) with slight modifications. Briefly, leaf tissue was extracted with 100 mM Tris–HCl buffer, 5 mM MgCl_2_, 2 mM EDTA-Na_2_, 5 mM DTT and 2% polyvinylpyrrolidone. The enzyme extract was incubated with 50 mM UDP-glucose, 50 mM fructose-6-P, 5 mM MgCl_2_, 2 mM EDTA-Na_2_, 5 mM DTT. The reaction was stopped using 100 μL of 2 mM NaOH at 100°C for 10 min. The solution was then cooled and mixed with 1 mL of 0.1% resorcin and 3.5 mL of 30% HCl, and then incubated at 80°C for 10 min. SPS activity was expressed as μg sucrose content⋅g^–1^⋅min^–1^ FW. The determination of SS activity was similar to that of SPS with the exception that fructose-dependent was substituted for fructose 6-P. Leaf soluble protein content was assayed using the bicinchoninic acid (BCA) method (Smith et al., 1985). Briefly, leaf tissue was extracted with 0.05 M phosphate buffered solution (PBS). The supernatant was incubated with BCA reagent at 37°C for 30 min. The solution was then cooled to room temperature, and the absorbance of the solution was read at a wavelength of 562 nm. Soluble sugar was extracted with 80% of ethanol. The extract was incubated with sulfuric acid-anthrone reagent at 90°C for 15 min. The absorbance of the solution was measured at a wavelength of 620 nm (Giannakoula et al., 2010). Prior to the leaf sampling, chlorophyll content was measured by using a SPAD-502 portable chlorophyll meter (Minolta, Japan). Three SPAD readings per leaf, and six plants in each plot were measured at the same place of leaf sampling. To each plot, the mean SPAD readings were calculated. Banana was harvested at maturity stage. Banana yield was determined by recording the weight of fresh banana using a loading balance and was calculated as mean yield per plant.

### Statistical Analysis

One-way ANOVA and Tukey’s test were used to evaluate the difference among treatments. Pearson linear correlations between the soil properties and leaf physiological attributes were determined. Factor analysis was performed for integrated evaluation of the impact of N application on soil properties and banana leaf physiological attributes. In the factor analysis, Principal component analysis (PCA) was used to extract factors from selected variance. It attempts to explain the complex variance with the minimum number of factors that better explain the variations. Factors are extracted in order of the weight of each factor. Factors with eigenvalues ≥1 of the variation in the data were retained (Brejda et al., 2000). Path analysis was used to evaluate the direct and indirect effects of soil and leaf factors on banana yield. Direct effects can be obtained from direct path coefficient. Indirect effects were calculated from the equation of path coefficient × correlation coefficient. Canonical correspondence analysis (CCA) was used to explore the important soil factors that can be responsible for the general changes of leaf physiological attributes. CCA was performed with “CANOCO” software, version 4.5. The significance of the variables was tested by Monte Carlo permutations (Luo K. et al., 2018). A cluster analysis based on Ward’s hierarchical method and Euclidean distances were used to group the seven treatments to investigate the similarities of soil and leaf variables response to N fertilization. SPSS 18.0 for windows was used for general statistical analyses. All values were expressed as means ± standard errors for three replicates.

## Results

### Effects of Nitrogen Fertilizer on Soil Properties

The influences of different N fertilizer application rates on soil properties are shown in Table 1. Soil enzyme activities were significantly affected by N fertilization treatments. With the exception of the N_5_ treatment, urease activities increased with increasing N rate up to 352 g (N_352_), and then decreased. The order of the soil urease activity was as follows: N_352_ > N_145_ > N_455_ > N_248_ > N_0_ > N_FT_ > T_CK_. There were no significant differences in soil urease activity among N_145_, N_352_ and N_455_, but were significantly higher than that in the control. A similar trend was observed for invertase activities, but the N_455_ treatment exhibited the highest activity. Invertase activity was in the order: N_455_ > N_352_ > N_248_ > N_145_ > N_FT_ > N_0_ > T_CK_. Compared with the control, significantly higher invertase activities were observed in the N_145_, N_248_, N_352_, N_455_, and N_FT_ treatments. In contrast to soil urease and invertase, acid phosphatase activities showed a decreasing trend with increasing N rate. The highest activity was observed in the N_0_ treatment, and the lowest in N_FT_. Significantly higher activities were observed in the N_0_ and N_145_ compared to other treatments.

**TABLE 1:** Soil properties of the banana field under different nitrogen fertilization rates.

**Treatment**	**Urease (mg⋅g^–1^ soil⋅24 h^–1^)**	**Invertase (mg⋅g^–1^ soil⋅24 h^–1^)**	**AP (mg⋅g^–1^ soil⋅24 h^–1^)**	**Available N content (mg⋅kg^–1^)**	**Available K content (mg⋅kg^–1^)**	**Available P content (mg⋅kg^–1^)**	**SOM content (g⋅kg^–1^)**
T_CK_	835.31 (54.51)d	3.49 (0.02)d	12.24 (0.40)c	36.17 (1.17)b	32.62 (1.56)f	25.74 (0.21)f	2.14 (0.05)a
N_0_	910.66 (18.65)cd	4.21 (0.22)cd	22.95 (0.63)a	46.08 (1.17)a	52.46 (1.95)c	57.36 (1.86)c	2.31 (0.08)a
N_14_	1171.57 (29.84)a	4.75 (0.26)abc	21.70 (0.55)a	49.58 (2.10)a	44.67 (1.16)e	173.04 (1.63)a	2.29 (0.04)a
N_248_	989.07 (25.31)b	4.91 (0.16)abc	17.93 (0.46)b	48.42 (0.58)a	86.26 (1.02)b	83.33 (1.36)b	2.13 (0.06)a
N_352_	1213.43 (29.58)a	5.20 (0.15)ab	12.47 (0.55)c	50.17 (1.17)a	103.26 (2.32)a	51.67 (0.21)d	2.33 (0.03)a
N_455_	1071.73 (16.31)ab	5.37 (0.02)a	13.62 (0.44)c	51.33 (1.54)a	67.73 (0.98)c	31.77 (0.34)e	2.30 (0.06)a
N_FT_	901.61 (12.78)cd	4.51 (0.04)bc	11.35 (0.53)c	46.67 (1.17)a	67.41 (0.78)d	28.45 (0.51)ef	2.19 (0.06)a

*Means (SE) followed by the different letters in a same column are significantly different at *p* < 0.05 (Tukey’s test). AP, Acid phosphatase; N, nitrogen; K, potassium; P, phosphorus; SOM, soil organic matter; T_CK_, control. N_FT_: chemical fertilization rate used by local farmers.*

The available N content showed an increasing trend with increasing N rate, with the exception of the N_FT_ treatment. The highest content was in the N_455_ and the lowest was in the T_CK_ treatment. There were no significant differences in soil available N content among the N fertilization treatments. In general, available K content exhibited an increasing trend with increasing N rate up to N_352_, and then decreased. The available K contents in all the treatments decreased in the following order of N_352_ > N_248_ > N_455_ > N_FT_ > N_0_ > N_145_ > T_CK_. The available K content in the N_352_ treatment was significantly higher than that in other treatments. Changes in available P content were similar to those of available K, and the highest content was in the N_145_ treatment. Available P content was in the order of N_145_ > N_248_ > N_0_ > N_352_ > N_455_ > N_FT_ > T_CK_. Although, no significant differences in SOM were observed for the different N fertilization rates, the highest SOM content was in the N_352_ treatment.

These results revealed that the response of soil properties to N fertilization varied differently. Different soil properties showed different optimal N application rates to reach its maximum values. When N rates were at relatively low levels, increasing N rate resulted in increasing trends in urease, invertase, available N, available K and available P. After reaching the optimal N rate, continue increasing N application caused decrease trend in these soil properties.

### Effects of Nitrogen Fertilizer on Leaf Physiological Attributes and Crop Yields

Leaf physiological attributes were affected by different N fertilizer application rate (Table 2). With the exception of the N_FT_ treatment, Nitrate reductase activities exhibited an increasing trend with increasing N rate. The highest activity was observed in the N_455_ treatment and was significantly higher than that in other treatments. The order of GS activities was as follows: N_352_ > N_455_ > N_FT_ > N_248_ > T_CK_ > N_0_ > N_145_. The highest GS activity was observed in N_352_ treatment. In general, SPS activities showed a decreasing trend with increasing N rate among the N fertilization treatments. The N_FT_ treatment had significantly lower SPS activity than that in other treatments. There were no significant differences in SS activity among the treatments. The highest SS activity was in the N_455_, followed by the N_352_ treatment.

**TABLE 2:** Leaf physiological attributes and yield of banana under different nitrogen fertilization rates.

**Treatment**	**NR (μmol⋅g^–1^⋅h^–1^)**	**GS (μmol⋅g^–1^⋅h^–1^)**	**SPS (μg⋅g^–1^⋅min^–1^)**	**SS (μg⋅g^–1^⋅min^–1^)**	**Soluble protein (mg⋅g^–1^)**	**Soluble sugar (mg⋅g^–1^)**	**Chlorophyll content (SPAD value)**	**Yield (kg)**
T_CK_	0.69 (0.03)ab	7.61 (0.39)cd	329.68 (22.52)bc	632.82 (37.79)a	38.64 (1.05)c	123.67 (2.27)d	56.83 (1.24)a	11.93 (1.33)b
N_0_	0.64 (0.00)b	7.60 (0.47)cd	547.02 (21.04)a	814.61 (58.91)a	35.29 (0.50)d	135.68 (1.88)d	57.73 (2.05)a	19.85 (0.99)a
N_145_	0.71 (0.03)ab	5.72 (0.25)d	373.42 (25.54)b	721.68 (45.83)a	39.57 (0.79)c	139.14 (3.74)c	57.83 (2.07)a	19.78 (1.70)a
N_248_	0.72 (0.03)ab	9.69 (0.50)bc	347.72 (16.02)bc	782.34 (51.31)a	41.06 (0.48)c	150.82 (3.79)bc	55.37 (2.77)a	19.39 (0.82)a
N_352_	0.71 (0.00)ab	13.45 (0.85)a	228.62 (4.73)d	831.52 (52.21)a	51.51 (0.16)a	186.31 (2.84)a	63.63 (0.37)a	21.83 (0.29)a
N_455_	0.78 (0.03)a	10.82 (0.45)b	277.87 (18.53)cd	833.12 (41.68)a	46.59 (0.22)b	162.95 (1.43)b	62.87 (1.07)a	21.47 (0.22)a
N_FT_	0.69 (0.03)ab	9.91 (0.44)bc	221.71 (16.49)d	777.90 (62.50)a	44.48 (0.95)b	124.43 (2.86)d	59.33 (2.37)a	18.37 (2.61)a

*Means (SE) followed by the different letters in a same column are significantly different at *p* < 0.05 (Tukey’s test). NR, nitrate reductase; GS, glutamine synthetase; SS, sucrose synthase; SPS, sucrose phosphate synthase; T_CK_, control. N_FT_: chemical fertilization rate used by local farmers.*

Soluble protein content increased gradually with increase in N rate and then decreased. The highest content was in the N_352_ treatment. Soluble protein content was in the order: N_352_ > N_455_ > N_FT_ > N_248_ > N_145_ > T_CK_ > N_0_. Variation in soluble sugar content was similar to that of soluble protein. The order of soluble sugar content in all treatments was as follows: N_352_ > N_455_ > N_248_ > N_145_ > N_0_ > N_FT_ > T_CK_. Chlorophyll content did not change significantly among treatments. The highest content was in the N_352_, followed by the N_455_ treatment. Crop yields showed a similar trend to chlorophyll content. There were no significant differences among N fertilization treatments.

Similar to the response of soil properties to N fertilization, at relatively low N levels, increasing N rate tended to improve NR, GS, SS, soluble protein, soluble sugar and chlorophyll values. After N rate reached a certain level, additional N had no significant effect on leaf physiological attributes or even led to a decrease.

### Integrated Assessment of the Effects of Different N Treatments on Soil and Leaf Physiological Factors

We performed factor analysis to group 14 soil properties and leaf physiological attributes into three factors explaining 86.17% of the total variance (Table 3). Factor 1 explained 41.67% of the variance with high positive factor loadings (>0.80) on invertase (0.81), available N (0.83), SS (0.93), and soluble sugar (0.83). This factor was associated with carbon and nitrogen metabolism in plants and thus was named the carbon and nitrogen metabolism factor. The treatment with the highest score (0.57) for factor 1 was N_352_. This indicated that the N_352_ was the most effective for improving carbon and nitrogen metabolism in banana. Factor 2 explained 27.72% of the variance with a high absolute factor loading (>0.80) on SPS (−0.95). Factor 2 was named the sucrose metabolism factor. The N_455_ treatment had the highest score (0.21) for factor 2. This revealed that the N_455_ treatment was dominant in increasing banana sucrose metabolism. Factor 3 explained 16.78% of the variance with high positive factor loading (>0.80) on available P (0.95) and was named the soil phosphorus nutrients factor. The N_145_ treatment had the highest score (0.35) for factor 3, indicating the N_145_ was the most effective at improving soil available P.

**TABLE 3:** The integrated score of different treatments.

**Treatment**	**Factor 1 score (order)**	**Factor 2 score (order)**	**Factor 2 score (order)**	**Integrated score (order)**
T_CK_	−0.73 (7)	0.07 (4)	−0.11 (6)	−0.91 (7)
N_0_	0.19 (3)	−0.59 (7)	−0.09 (5)	−0.57 (6)
N_145_	−0.12 (5)	−0.05 (6)	0.35 (1)	0.20 (3)
N_248_	−0.06 (4)	0.04 (5)	0.06 (2)	0.05 (4)
N_352_	0.57 (1)	0.20 (2)	−0.08 (4)	0.81 (1)
N_455_	0.30 (2)	0.21 (1)	0.01 (3)	0.61 (2)
N_FT_	−0.15 (6)	0.12 (3)	−0.14 (7)	−0.19 (5)

*T_CK_: control; N_FT_: chemical fertilization rate used by local farmers.*

**FIGURE 1:**
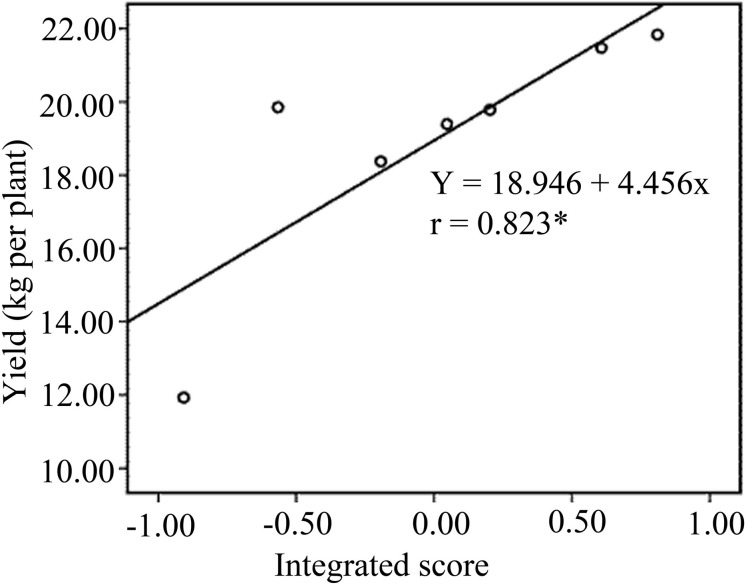
Linear regression relationship between integrated score and banana yields of treatments.

The N_352_ treatment had the highest integrated score among all treatments, followed by N_455_. The T_CK_ treatment exhibited the lowest integrated score. The integrated score for all treatments was as follows: N_352_ > N_455_ > N_145_ > N_248_ > N_FT_ > N_0_ > T_CK_. Further correlation analysis revealed significant and positive correlations between integrated score and crop yield under different N treatments (Figure 1).

### Effects of Soil and Leaf Physiological Factors on Crop Yields

We assessed the direct and indirect effects of soil and leaf factors on crop yields using path coefficient analysis. As shown in Table 4, among soil properties, invertase exhibited the highest direct positive effect (0.76) on crop yields, followed by SOM (0.70), available P (0.53), available K (0.47), phosphatase (−0.06), and urease (−0.85). These results suggested that invertase and SOM (>0.70) were the main factors for improving the crop yields.

**TABLE 4:** Direct and indirect path coefficient values of soil and leaf factors on crop yields.

	**Urease**	**Invertase**	**AP**	**Available K**	**Available P**	**SOM**
Urease	−**0.851**	0.58	−0.01	0.24	0.28	0.46
Invertase	−0.66	**0.761**	0.00	0.35	0.10	0.35
AP	−0.10	−0.03	−**0.058**	−0.13	0.37	0.20
Available K	−0.44	0.56	0.02	**0.469**	−0.08	0.14
Available P	−0.45	0.15	−0.04	−0.07	**0.532**	0.14
SOM	−0.56	0.38	−0.02	0.09	0.11	**0.704**
	NR	GS	SPS	SS	soluble sugar	chlorophyll
NR	−**0.167**	−0.41	0.34	0.26	0.34	−0.04
GS	−0.08	−**1.046**	0.37	0.74	0.52	−0.07
SPS	0.01	0.67	−**0.580**	−0.06	−0.23	0.05
SS	−0.15	−0.66	0.03	**1.183**	0.45	−0.06
Soluble Sugar	−0.12	−0.80	0.20	0.77	**0.683**	−0.07
Chlorophyll	−0.09	−0.75	0.31	0.69	0.48	−**0.096**

*Bold numerals are the direct effect values. AP, acid phosphatase; K, potassium; P, phosphorus; SOM, soil organic matter; NR, nitrate reductase; GS, glutamine synthetase; SS, sucrose synthase; SPS, sucrose phosphate synthase.*

With respect to leaf physiological attributes, the direct positive effect on crop yields decreased in the following order: SS (1.18) > soluble sugar (0.68) > chlorophyll (−0.10) > NR (−0.17) > SPS (−0.58) > GS (−1.0). These findings indicated the relative importance of leaf attributes on crop yields.

The indirect path coefficients revealed the degree that the soil and leaf factors, to a certain extent, contributed to crop yields through affecting the other factors.

### Impact Factors and Contributions of Soil Properties to Leaf Physiology

**FIGURE 2:**
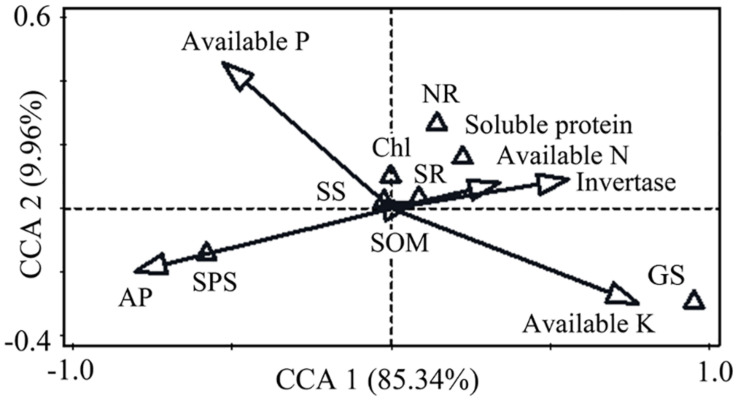
Canonical correspondence analysis (CCA) of the relationship between soil properties and leaf physiological attributes. Soil factors are represented by hollow arrows. Leaf attributes are represented by hollow triangle. The first axis (horizontal) and the second axis (vertical) explained 85.34 and 9.96% of the variation. SR, soluble sugar; Chl, chlorophyll.

In order to comprehensively evaluate the relationship between soil properties and leaf physiological attributes, we evaluated the impacts of soil properties on leaf attributes by CCA analysis (Figure 2). Monte Carlo tests revealed that soil acid phosphatase, invertase, available K and available P were strongly related to leaf physiological attributes. The first and second ordination axes explained 95.3% of the total variation. Acid phosphatase (56.1, *p* = 0.08), available K (52.3, *p* = 0.10), invertase (27.3, *p* = 0.22), and available P (26.7, *p* = 0.21) were strongly correlated with leaf physiological attributes. Available K and invertase were positively correlated with the first axis, while a negative correlation was observed between acid phosphatase and the first axis. The results of mantel test indicated that available K, invertase, acid phosphatase and available P were the four most important soil properties affecting the variations in leaf physiological attributes.

### Cluster Analysis of Soil Properties and Leaf Physiological Attributes

**FIGURE 3:**
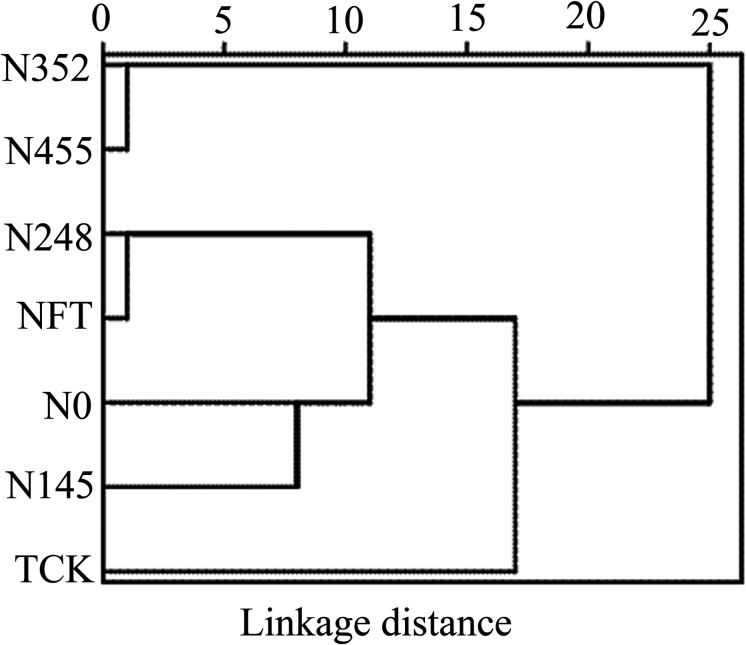
Cluster analysis (Euclidean distances, Ward’s hierarchical method) based on soil and leaf physiological factors of different treatments.

We performed a cluster analysis to identify similarities of the responses to N fertilization on soil properties and leaf physiological attributes among different N treatments (Figure 3). The N_352_ and N_455_ treatments were grouped together as a separate cluster. The T_CK_ treatment was grouped into one category alone. Although the N_248_, N_FT_, and N_0_ treatments were grouped in the same main cluster, the N_248_ and N_FT_ treatments branched more closely to each other than the N_0_ treatment.

### Correlations Between Soil Properties and Leaf Physiological Attributes

There were significant and positive correlations between soil properties and leaf physiological attributes (Table 5). Leaf GS was significantly and positively correlated with available K (*r* = 0.869, *p* < 0.05). Leaf SPS was significantly and positively correlated with acid phosphatase (*r* = 0.868, *p* < 0.05). Leaf SS was significantly and positively correlated with invertase (*r* = 0.782, *p* < 0.05) and available N (*r* = 0.819, *p* < 0.05). Leaf soluble protein was significantly and positively correlated with available K (*r* = 0.759, *p* < 0.05). Leaf soluble sugar was significantly and positively correlated with soil urease (*r* = 0.787, *p* < 0.05), invertase (*r* = 0.790, *p* < 0.05) and available K (*r* = 0.799, *p* < 0.05).

**TABLE 5:** Pearson’s correlation coefficients between soil properties and leaf physiological attributes.

	**Urease**	**Invertase**	**AP**	**Available N**	**Available K**	**Available P**	**SOM**
NR	0.47	0.68	–0.38	0.47	0.31	–0.01	0.06
GS	0.347	0.59	–0.63	0.40	0.87*	–0.54	0.20
SPS	–0.28	–0.39	0.87*	–0.17	–0.46	0.30	0.15
SS	0.44	0.78*	0.08	0.82*	0.73	–0.14	0.60
Soluble protein	0.60	0.70	–0.67	0.51	0.76*	–0.27	0.31
Soluble sugar	0.79*	0.80*	–0.15	0.65	0.80*	–0.00	0.55
Chlorophyll	0.58	0.62	–0.47	0.51	0.49	–0.31	0.70

*Correlation is significant at the 0.05 level (Two-tailed). AP, acid phosphatase; N, nitrogen; K, potassium; P, phosphorus; SOM, soil organic matter; NR, nitrate reductase; GS, glutamine synthetase; SS, sucrose synthase; SPS, sucrose phosphate synthase.

## Discussion

Among environmental factors that influence crop growth, soil properties play a fundamental role. The soil properties can be modified by N fertilization. Soil enzyme activity reflects the intensity of biochemical processes occurring in soil and reflecting the soil nutrient supply capacity. Therefore, enzyme activities correlate closely with soil fertility (Stark et al., 2008). Urease hydrolyzes urea to carbon dioxide and ammonia, and affects N uptake of crops. Soil phosphatase hydrolyzes P from SOM to inorganic P which can be utilized by plant (Burns et al., 2013). Soil invertase hydrolyzes sucrose to glucose and fructose, and is involved in the carbon cycle. In our study, urease and invertase activities increased with increasing N application. This is in agreement with other reports (Wang et al., 2008; Liang et al., 2014). However, continued increase in N rate caused a decrease in urease activity, suggesting that excessive N appliation suppressed urease activity. We found acid phosphatase ativity decreased with increasing N rate. In contrast, Allison et al. (2006) had shown an increase in acid phosphatase activity with N fertilization. This could be attributed to the differences in soil type and soil properties.

Soil microorganisms are involved in soil nutrient mineralization and cycling, and thus affect soil available nutrient contents. Previous studies have documented the impact of N fertilization on soil microbial composition and functions (Ramirez et al., 2010; Lupwayi et al., 2012; Zhang et al., 2017). Excessive N fertilization suppresses the richness and diversity of soil microbes (Wei et al., 2018). In addition, high N rates result in vigorous plant growth and higher nutrient uptake from soil. Consequently, soil available nutrients are decreased. Mineral fertilizer application could increase SOM content by returning crop residue to the soil. Meanwhile, C mineralization leads to a decrease in SOM content. Therefore, SOM contents maintain a dynamic equilibrium in soil. In this study, increased N fertilization had no significant influence on SOM content, which is in agreement with other studies (Brown et al., 2014; Zhang et al., 2015b).

Nitrate reductase and GS are key enzymes involved in N assimilation and metabolism (Heidari et al., 2011; Thomsen et al., 2014). NR is a substrate inducible enzyme whose induced activity is regulated by nitrate availability. In the present study, NR activity increased with increasing N application rate. This may be due to more available nitrogen under high N conditions (Vijayalakshmi et al., 2015). The present research showed that the highest GS activity was observed at the N6 rate and then decreased significantly with increasing N rate. This indicates that there is a threshold of N fertilization rate for inducing the highest GS activity. Sucrose metabolism and transport are closely related to crop yield. SS and SPS are key enzymes involved in sucrose metabolism. The present results showed a decreasing trend of SPS activity, possibly due to the conversion of sugars to starch (Tung et al., 2018).

Soluble sugars are the primary products of photosynthesis, and their content reflects the rates of carbohydrate metabolism rates. In this study, when N rate was at a relatively low level, increasing N rates had a positive influence on soluble sugar content. This may be a result of better carbon assimilation. However, the synthesis of carbohydrates was inhibited under relatively high N rate. Many enzymes are present in the form of soluble proteins and are involved in photosynthesis. In the present study, N application increased soluble protein content. Similar to soluble sugar content, excessive N application was not beneficial for improving soluble protein content. Nitrogen is closely associated with chlorophyll synthesis. Although no significant differences were observed in chlorophyll content among N treatments, the highest content was observed in N_352_ and the further increase in N rate decreased chlorophyll content.

Determining the optimal N application rate is an effective method to meet crop economic benefits while reducing the environmental risks. Crop yield is a commonly used indicator for determining optimal N application rate (Wang et al., 2017). In this study, there were no significant in banana yield among N treatments. However, the maximum yield was observed with N_352_. Furthermore, the N application rate in N_352_ was far less than that in N_FT_. These results indicated that N rates above 352 g per plant exceed the optimal N application. Previous studies have shown that when N application rates exceed a threshold, increasing N rate has no significant effect on increasing crop yield (Liu and Wiatrak, 2012; Qiao et al., 2012; Li et al., 2019). This is similar to the results in our study.

In this study, factor analysis revealed that N_352_ treatment had the highest integrated score in all treatments. This indicated that the 352 g per plant is the optimal N rate for improving banana soil and physicochemical factors. Although, the N_FT_ treatment did not rank lowest for integrated score, it had the lowest factor 1 and factor 3 scores. This indicated that N_FT_ treatment had a greater negative effect on carbon, nitrogen metabolism and soil phosphorus nutrients than other treatments. Linear regression showed that integrated score was significant and positive linear correlation with banana yield. Therefore, developing coordinated and integrated N management practices including soil fertility and crop physiological conditions would be beneficial to banana yield. Path analysis revealed that invertase, SOM and SS plays the most important role in improving banana yield in this study. The results also indicate that the improvement of these soil and physiological attributes will improve banana yield.

Soil enzymatic activities affect the mineralization of soil nutrients such as N, P and K. These soil available nutrients are absorbed and utilized by plants. Thus, there is a close relationship between soil enzyme activities, soil nutrients and plant physiological attributes. The present study also revealed positive correlations between these soil properties and leaf physiological attributes. The CCA results further revealed the ecological relationships between soil properties and plant growth requirements comprehensively, and suggested a nutrient management strategy with pertinence. According to cluster analysis, there were distinct separations among different treatments in this study. This revealed that soil properties and leaf physiological attributes were obviously affected by N application treatment, and there was high similarity between the N_352_ and the N_455_, the N_248_ and the N_FT_ treatments.

Carbon/nitrogen rate and sucrose/amino acids ratio are also important leaf physiological attributes and play important roles in yield. Therefore, future work is still needed to identify the effect of different N fertilization rates on C/N rate and sucrose/amino acids ratio of banana leaf.

This study demonstrated that excessive application of N fertilizer had a negative effect on banana growth and soil fertility. To benefit banana agronomy and environment, the N application rate of 352 g N per plant could be suggested as the optimal N application rate in this region.

## Conclusion

We demonstrated that different N application rates had great impacts on soil properties and banana leaf physiological attributes. Excessive N fertilization had a negative effect on soil and leaf physiological factors. When simultaneously considering banana yield, physiological conditions and soil fertility, the recommended optimal N application rate is 352 g N per plant in this region. Invertase, SOM and SS were the dominant factors for improving crop yield. Soil available K, invertase, acid phosphatase and available P were the most important factors related to leaf physiological conditions. Our results suggest that reducing N application rates is an effective approach for improving soil fertility and crop yield.

## Data Availability Statement

The original contributions presented in the study are included in the article/supplementary material, further inquiries can be directed to the corresponding author/s.

## Author Contributions

MP and JS conceived and designed the experiments. JS, CL, and WL performed the experiments. WC and SZ analyzed the data. JS wrote the manuscript. YZ and CZ reviewed and edited the manuscript. All authors read and approved the manuscript.

## Conflict of Interest

The authors declare that the research was conducted in the absence of any commercial or financial relationships that could be construed as a potential conflict of interest.
